# Lateralized Effects in Troxler Fading and Parvo and Magnocellular Processing Tasks after Localized 1Hz rTMS

**Published:** 2021-06-15

**Authors:** Patricia A. Taylor-Cooke, Joseph G. Chacko, Kenneth Chelette, Mark S. Mennemeier

**Affiliations:** 1 Department of Neurobiology and Developmental Sciences, University of Arkansas for Medical Sciences, USA; 2 Departments of Ophthalmology and Neurology, University of Arkansas for Medical Sciences, USA; 3 ANT Neuro North America, USA

**Keywords:** troxler fading, repetitive transcranial magnetic stimulation, rTMS, 1Hz

## Abstract

Troxler Fading (TF) is a complex visual phenomenon with uncertain mechanisms. This study was performed to test hypotheses concerning the contributions of parvocellular and magnocelluar processing in extrastriate pathways to TF. The study used low-frequency, repetitive Transcranial Magnetic Stimulation (rTMS) delivered at target sites in the parietal, temporal and dorsolateral frontal cortex to alter performance on a TF paradigm and on tests sensitive to parvocellular and magnocellular processing. Nine, right-handed, healthy subjects completed 3 tasks, TF, Texture Detection (TD), and Motion Detection (MD), at baseline and after undergoing 15 minutes of low-frequency rTMS at each cortical site on separate occasions. Results revealed lateralized effects of rTMS on each test. Left temporal stimulation slowed the parvocellular, TD task and it accelerated TF. Right parietal stimulation markedly accelerated TF whereas left parietal stimulation slowed TF. Right frontal stimulation accelerated performance on the magnocellular, MD task. Taken together and in the context of other research studies, the findings suggest hemispheric specialization both for TF and for the parvocellular and magnocellular processing tasks.

## Introduction

A variety of models have been proposed historically to describe the complexity of visual processing pathways; their interconnections and feedback mechanisms [1–6]. In general, they posit that visual information originates from the ganglion cells of the retina and project along two main parallel paths [magnocellular (MC) and parvocellular (PC)] to specific regions within the lateral geniculate nucleus (LGN) of the thalamus and then continue to the primary/striate cortex of the occipital lobe (area V1; Brodmann’s area 17). Each pathway maintains distinct retinotopic maps up to this point which then diverge from the primary visual cortex into separate extrastriate cortical regions where distinct anatomic segregation of the PC and MC pathways may be lost among the interconnections, duplications and feedback projections [7,8]. More recently, however, a high-resolution fMRI study in humans showed segregated MC and PC processing pathways in four areas of the extrastriate cortex suggesting that MC and PC information may be processed in separate parallel channels throughout much of the human visual system and not only in the earlier stages of the visual system [9].

Beyond primary visual cortex two extrastriate processing streams are recognized as the ventral “what” and the dorsal “where” pathways. The what pathway proceeds from area V1 to areas V2/V3 and V4 towards the inferior temporal cortex [7]. This projection system is considered to be more specialized for object identification [2]. Its physiological properties are specialized for high spatial resolution, low temporal resolution, and color [4,5]. In contrast, the where pathway proceeds from area V1 to V5 and towards the posterior parietal cortex. The where pathway is considered to be more specialized for processing motion perception [2,7]. Its physiological properties are specialized for low spatial resolution and high temporal resolution [4] and fast, low contrast motion [5]. Importantly, both pathways have the capability to process information in both spatial and temporal domains, but each pathway extends visual processing along one specific domain [5]. Both pathways also receive input from the dorsalateral prefrontal cortex [8,11]. Tootell and Nasr [9] found MC and PC functions and connections in cortical columns in V2,V3 and V4 indicating that these processing streams exist and extend through the early and middle stages of extrastriate cortex in humans.

Transcranial magnetic stimulation (TMS) has been used to investigate a variety of different types of visual processes. TMS induces electrical stimulation of cortical neurons by creating a brief, focused magnetic field over the surface of the brain. When magnetic pulses are delivered repetitively and rhythmically, the process is called repetitive TMS (rTMS). Approximately, 1–3 cm of tissue is stimulated beneath the coil depending on coil configuration [12] and the magnetic field declines rapidly with distance away from the coil [13,14]. Thus, current TMS coils are only able to directly stimulate the superficial cortex, but deeper brain structures and distant regions of cortex may be affected by rTMS via cortical–subcortical and cortical-cortical connections.

Historically, the effects of TMS have been considered to be excitatory or inhibitory and they have been evaluated at different points in time following stimulation [15]. Immediate effects are thought to result from direct excitation of inhibitory or excitatory neurons, such as a muscle twitch immediately following stimulation. Intermediate effects of TMS are those occurring minutes after stimulation. For example, low-frequency repeated stimulation of a single neuron produces long-lasting inhibition of cell–cell communications [16,17] whereas high-frequency stimulation can improve cell–cell communication [18]. Even though TMS stimulates hundreds to thousands of neurons, early studies of the motor cortex have showed that low-frequency stimulation (≤ 1Hz) tended to produce an inhibitory intermediate effect [19] whereas high-frequency stimulation (>5Hz) tended to produce excitatory intermediate effects [20,21]. Studies combining rTMS and functional neuroimaging suggested that low-frequency rTMS reduced cortical excitability, both locally and in functionally linked cortical regions through synaptic transmission [22–25]. Later studies revealed variability in these general effects both within and between subjects but 1Hz rTMS is believed to have an inhibitory effect that lasts for minutes after the period of actual rTMS delivery.

Transcranial magnetic stimulation has also been used to study visual perception. Low frequency rTMS has been used to disrupt cortical processing temporarily in normal subjects, sometimes referred to as a “virtual lesion”. For example, motion detection can be disrupted by inhibiting parietal cortex with low frequency rTMS – causing deficits contralateral to stimulation [26]. Low frequency rTMS over right parietal cortex can simulate both left neglect, inattention to visual stimuli [27,28], and extinction, disrupting target identification contralateral to stimulation when targets are presented to both spatial hemifields [29]. Finally, low frequency rTMS over right frontal and right parietal cortex led to deficits consistent with motor-intentional and sensory-attentional neglect, respectively [30]. Research also suggests neurons in the superior temporal sulcus are involved in motion processing [31] and neurons in parietal cortex are involved in visual search [32]. However, it is uncertain from rTMS studies whether these areas actually process a particular type of information versus whether rTMS merely affects processing by disrupting interconnections between these areas.

The current study sought to use low frequency rTMS to selectively disrupt processing on tasks that should be sensitive to MC processing like motion detection (MD) and to PC processing like texture discrimination (TD). Tasks used in this study were developed on the basis of Schiller and colleagues [5] work in the monkey to examine MC and PC processing. Schiller’s work showed that TD is mediated exclusively within the PC system and MD relies on the MC pathways. fMRI studies were examined to help guide the placement of the TMS coil so as to disrupt processing on these tasks selectively. Several areas within the inferior occipito-temporal cortex (i.e., the what pathway) become activated by form and shape, and so should be sensitive to a TD task, but two areas were strongly activated. The largest is the posterior inferior temporal gyrus (post-ITG). A second, smaller region is in the middle fusiform gyrus [33], corresponding to Brodmann areas 37 and 19, respectively. The middle fusiform gyrus is too deep in the brain to be reached by conventional TMS; therefore, the posterior aspects of the middle and inferior temporal gyri were targeted in our study as these areas are accessible to rTMS.

Functional MRI studies have also attempted to identify cortical areas involved in MD. Motion detection is mediated primarily within the where pathway. Functional MRI studies that examined brain activity while viewing dots that move at random found activation at the junction of the intraparietal sulcus and the parieto-occipital sulcus and at the posterior intraparietal sulcus. Because the junction of the intraparietal and parieto-occipital sulcus is known to be a convergence zone [34], where input from MC and PC pathways combine the posterior aspect of the intraparietal sulcus was targeted for rTMS in the current study to more selectively interrupt MD.

This study also examined how rTMS disrupted performance on a task known as Troxler Fading (TF) - the fading of an image in peripheral vision during steady fixation. Without using image stabilization techniques, TF occurs over 10s of seconds and it is disrupted by movement of an image across the peripheral retina. The primary reason for examining TF in this study was a hypothesis proposed by Livingston and Hubel [4] decades ago that image fading must occur within a pathway sensitive to PC processing such as the what pathway because processing in a pathway sensitive to MC processing occurs too quickly to account for fading over 10s of seconds. If this hypothesis were true, then TMS-induced disruptions of TF should occur at the same location as rTMS induced disruptions of TD but not MD. An alternative hypothesis is that TF may depend on intercommunication between pathways in both systems such that fading within the system is sensitive to parvocellular processing might be “canceled” by detection of motion in the pathway sensitive to magnocellular processing. If this hypothesis is true, then performance on MD tasks should share variance with that on TF.

Toward the latter hypothesis, a preliminary behavioral study indicated that while performance on TD task was the best predictor of performance on a TF task, a model that included performance on a MD task improved prediction [35]. The current study aimed to go further by examining disruptions in TF following low-frequency rTMS disruption in the target sites described above, those activated by form/shape and movement, respectively.

Preliminary studies also examined processing asymmetries across the peripheral retina. Earlier work had shown that TF is faster on the vertical than horizontal visual meridian [35–37]. Our behavioral study uniquely showed that processing on TD and MD tasks is similarly asymmetric across the peripheral retina. Opposite TF, however, both TD and MD are faster rather than slower on the horizontal than vertical meridian. The difference between TF and the TD and MD tasks may simply be due to the nature of these tasks. Whereas TF involves habituation, which is relatively preserved along the horizontal meridian, the PC and MC tasks involve detection which is faster along the horizontal meridian [35]. The asymmetries in each case might merely be viewed as processing efficiencies along the horizontal meridian but they might also indicated that TF is related to parvocellar processing in a way suggested by Livingston and Hubel [4].

Both the what and the where pathways receive input form DLPF cortex and lesions of the DLPFC disrupt TF. The DLPF is an important component of the attentional systems that modifies sensory experience [38–40]. Studies of TF involving patients with brain injury showed that lesions to DLPF cortex inhibit and even prevent TF; whereas, lesions in parietal cortex accelerate image fading [41]. These results revealed an important influence of attention on image fading. Parietal cortex appears to work to preserve attention for visual percepts; whereas attentional processes mediated by the frontal lobes work to habituate processing for redundant sensory information (such as the peripheral image during TF). For this reason, low-frequency TMS was also delivered to DLPF to determine how it contributes to performance on the TF, TD, and MD tasks. The hypotheses for the current study were as follows: 1) if TF occurs exclusively within a system sensitive to parvocellular processing, then rTMS that disrupts PC processing (e.g., posterior temporal; Brodmann’s area 37) will cause accelerated TF contralateral to stimulation because the PC system will be effectively impaired and any resistance it offers to image fading will be compromised. In contrast, if TF depends on systems sensitive to both PC and MC processing, then rTMS that disrupts MC processing (posterior parietal; Brodmann’s area 7) will also cause accelerated TF contralateral to stimulation because the MC system will fail to interrupt fading via motion detection. (Here we are referring to the ability to detect motion due to image movement across the retina due to micro and corrective saccades during fixation which normally resist TF). Third, rTMS disruption of the DLPF (Brodmann’s area 46) will either retard or prevent TF because it will impair habituation. Conversely, the same disruption will render processing on the TD and MD tasks more efficient (faster detection) because they will release these systems from frontal lobe inhibition.

## Method

### Participants

Nine right-handed, healthy subjects (two women, six men, age range=22 to 27, *M* age =23.5) were recruited in an Institutional Review Board (IRB) approved study for use with human subjects, in accordance with the ethical principles of the 1964 Declaration of Helenski. All subjects underwent an informed consent process and provided written consent before participation in study procedures. See Table 1 for subject demographics.

All subjects had normal MRI scans as reviewed by the study physician (JC), a neuro-ophthalmologist. Exclusion criteria included a history of neurological illness, uncontrolled medical conditions, epilepsy, severe psychiatric disorders, metal implants in the head or neck, current medications known to lower seizure threshold, prior head injuries, migraines, drug dependency or abuse, and eyeglasses that were bifocal, trifocal, multifocal or monovision.

### Apparatus and materials

All programs were created using the Visual Stimulus Generator (VSG2/4F; Cambridge Research Systems) and the stimuli were delivered on a Sony Trinitron (Multiscan 17seII) color monitor. Stimulus presentation was controlled by a GP7–500 Gateway computer. All responses were made on a response box connected directly to the computer. A Black and White Mini Camera (BD-108W-X), which can be used to view in darkness down to 0.003 Lux, was used to monitor fixation. Visual diagnostics was conducted with the Pelli-Robson Contrast Sensitivity Chart from HS Clement Clarke International (1988) and a visual acuity card to assess acuity for the distance of the monitor from the subjects’ eyes. The Wechsler Adult Reading Test (WTAR), shown to be a valid estimate of IQ, was used to estimate IQ.

### Tasks

#### Troxler fading

The visual stimuli are dots subtending 0.23 degrees of visual angle. Dots were presented in peripheral vision at 19.61 degrees of visual angle. Dots are gray (luminance=14.6 cd/m^2^), the central fixation point was yellow (luminance=96.71 cd/m^2^), and the background screen was dark gray (luminance=0.57 cd/m^2^). Dots appeared in random order across the eight visual locations and remained until the subject responded or the time-out limit (30 seconds).

#### Texture detection

The visual stimulus was a field of dark gray, diagonal dashed lines (luminance=0.81 cd/m^2^) presented on a light gray background (luminance=71.62 cd/m^2^) with a red crosshair for fixation (luminance=21.28 cd/m^2^). One of the eight locations will have dashed lines going in the opposite direction, which subtends 3.75 degrees of visual angle and was presented at 14.85 degrees of visual angle. Stimuli fade in at a constant rate from a blank screen. Subjects were asked to press the response button once they have detected the location of the target and then name the location.

#### Motion detection

The visual stimulus was a screen of black random dots (luminance=0.1 cd/m^2^), subtending 0.23 degrees of visual angle, on a gray background (luminance=4.30 cd/m^2^) with a yellow fixation crosshair (luminance=96.71 cd/m^2^). During trials dots were presented at 14.85 degrees of visual angle. Random dots will appear all at once. In one of the eight locations a group of dots will begin to move slowly and increase in distance of movement (pixels per second) at a constant rate until the location is detected.

#### Reaction time

The visual stimulus is a light gray dot (luminance=71.62 cd/m^2^) subtending 1.15 degrees of visual angle, on a black background (luminance=0.1 cd/m^2^). The stimulus was briefly presented at randomized variable rates (one to five second intervals) in the center of the screen.

### Procedure

Subjects completed eight testing sessions (Baseline, six rTMS sessions, and a follow-up session). All sessions were separated by at least 48 hours. Tasks and rTMS locations were counterbalanced across subjects.

Tasks were administered in a darkened room with a black shield surrounding the monitor to prevent glare and distraction from the examiner’s monitor and movement. Subject’s heads were secured in a head and chin rest 30.48 cm from the computer screen, which was parallel to their eyes and level with the pupils. All trials were randomly presented in blocks of eight trials and participants responded by pressing a button on a response box. The number of trials used during the baseline session was determined based on pilot study results. All tasks were randomly presented in blocks of eight trials for the eight peripheral locations (e.g., North, Northeast, East etc). Baseline testing was conducted to familiarize subjects with tasks by completing eight practice trials and a specified number of test trials on each task as follows: 1) TF - 40 test trials, 2) TD - Stimuli fade in at a constant rate from a blank screen. Subjects were asked to press the response button once they have detected the location of the target and then name the location. A total of 48 test trials were administered, 3) MD - Random dots will appear all at once. In one of the eight locations a group of dots will begin to move slowly and increase in distance of movement (pixels per second) at a constant rate until the location is detected. Forty test trials were administered. The follow-up session was the same as baseline. Trials were recycled when clear breaks in fixation occurred.

On the days subjects underwent rTMS, subjects completed four practice trials of each task to refamiliarize them with the tasks just prior to determining motor threshold. Immediately following the 15 minutes of rTMS each subject completed 16 test trials of each task followed by 30 RT trials.

#### rTMS

T1 weighted MRI scans were obtained using GE scanners at UAMS. A neuronavigational system (Brainsight Frameless Stereotaxy, Rouge Research) was used to guide the application of rTMS to the selected neuroanatomical regions. The Brainsight system allows real-time visualization of the coil in relation to a cortical area targeted for treatment on the MRI scan, which is downloaded as a DICOM file into the Brainsight system. A MagStim 200 series TMS machine with an air-cooled 70-mm figure-of-eight coil will be used to deliver stimulation (MagStim Co., UK). The motor threshold (MT) was determined by placing the TMS coil over the cortical motor area and delivering single pulses of increasing intensity until the optimal area of stimulation is found. Threshold was defined as the percentage of the maximum stimulator output necessary to elicit a motor evoked potential (MEP) of 50 μvolts recorded from the thenar muscle of the contralateral hand in 3 of 6 stimulus trials. MEPs were recorded with AgCl surface electrodes fixed on the skin with a belly-tendon montage. The EMG signal is filtered (10Hz–1kHz bandpass) and displayed on a computer screen.

Cortical stimulation sites were identified by neuroanatomical markers to ensure correct localization for each subject and avoid stimulating different sites due to individual variability in neuroanatomy. The location identified to interrupt MC processing was the dorsal lips of the posterior end of the intraparietal sulcus, which was located by finding the intraparietal sulcus, following it to the posterior end, and setting the target location on the dorsal lips. The location identified for PC processing was the posterior temporal lobe, which was located by finding the temporal lobe in each individual, setting the stimulation target approximately 1.5 cm anterior to the occipital lobe and 1.5 cm inferior to the superior most aspect of the temporal lobe at this location. The location identified for interrupting attentional mechanisms was Brodmann’s area 46, which was located by finding the middle third of the mid-frontal gyrus and centering the stimulation target in this region. The Brainsight system was used to position the coil directly over the identified cortical location. Each subject received 15 minutes of 1Hz rTMS (low frequency; 900 stimuli per session) at 110% of the subject’s motor threshold. Subjects then completed behavioral tasks for 15 minutes following stimulation. Convention holds that 15 minutes of stimulation at this intensity and frequency should produce inhibition that lasts for at least 8 to 10 minutes. A neuro-ophthalmologist trained in the safety precautions for TMS was on site for safety monitoring.

#### Data analysis

All analyses were conducted after visual task scores were transformed into z scores (*M=*0*, SD=*1) to avoid contamination of the results by between-subjects variability. Since there appeared to be a learning effect across sessions on the task performance, the z scores were regressed on session number to obtain a predicted score. The predicted score was then subtracted from original z scores to obtain residuals. The residualized performance scores were then used in the analyses, which serve to remove linear changes across sessions. Specific contrasts were selected to address each of the hypotheses and limit the number of analyses conducted to prevent the need to adjust for multiple comparisons. To compare the changes occurring in contralateral hemispace across tasks a change score from baseline was used for each task. The main interest was contralateral hemispace, which consists of three peripheral locations. Therefore, scores were collapsed across the three locations to obtain one average score to use for contralateral hemispace performance. Change scores were then compared using the specific contrasts to assess differences, particularly in opposite directions, as this was the expected directions for each hypothesis. For instance, For left stimulation a contrast was used to assess a collapsed score on northeast, east, and southeast locations across the two tasks. Fixation was monitored during task performance and only trials with sustatined fixation were analyzed.

## Results

Change scores from baseline to post-stimulation performance after left and right hemisphere stimulation at each target location were compared to test the hypotheses. Results indicated a difference in contralateral hemispace for TF and texture detection performance after left temporal stimulation [F(1,16)=4.93, *p*<0.05], but not after right temporal stimulation (*p*>0.05). Left temporal stimulation led to slower texture detection (PC processing; *M*=−0.66, *SE*=0.68) and faster TF performance (*M*=1.47, *SE*=0.68). Negative scores indicate slower performance after stimulation, whereas positive scores indicate faster performance. No other differences were found when comparing contralateral performance between texture detection or motion detection and TF for the other hypotheses or cortical locations.

**Figure 1.**
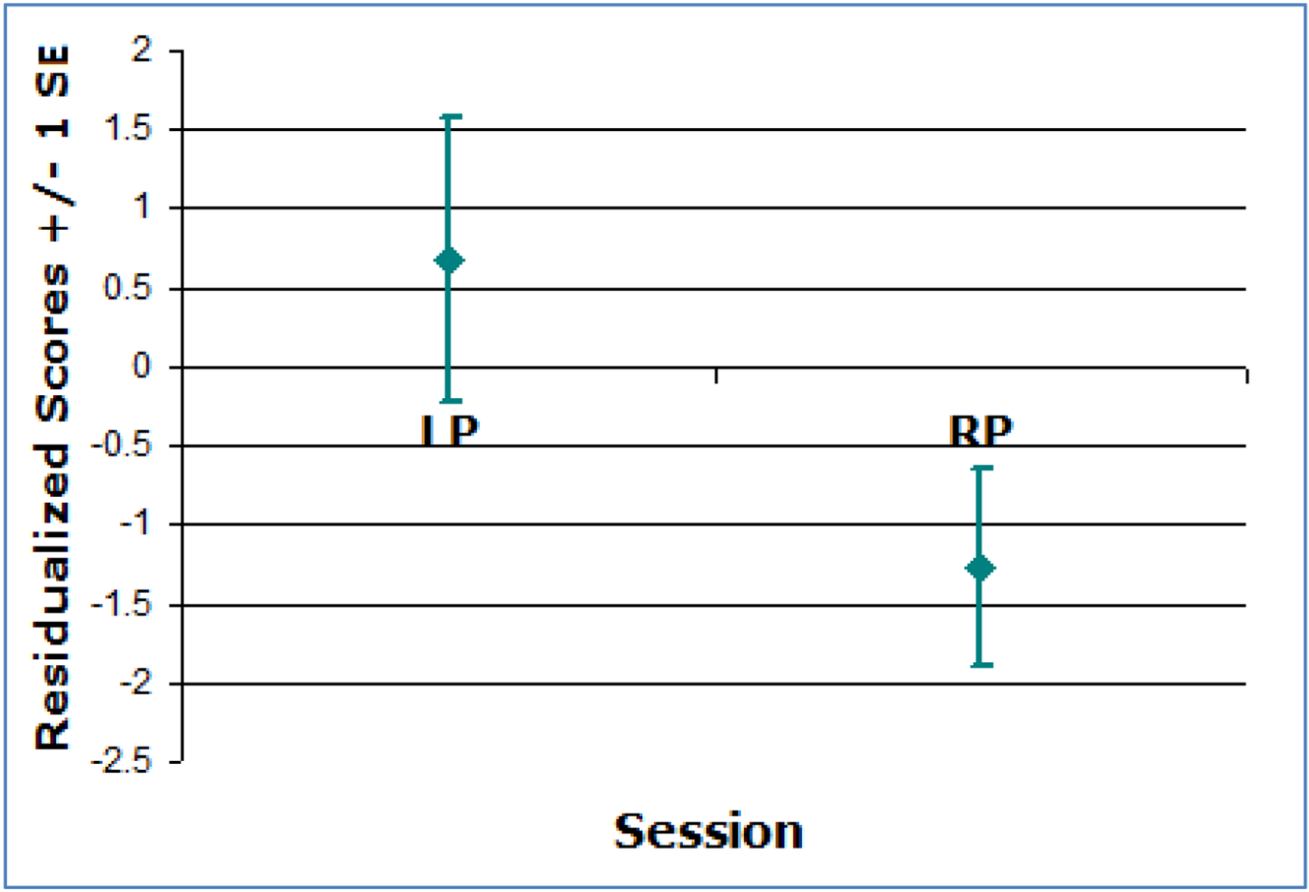
TF residualized performance scores averaged across all eight peripheral locations after right and left rTMS

**Figure 2.**
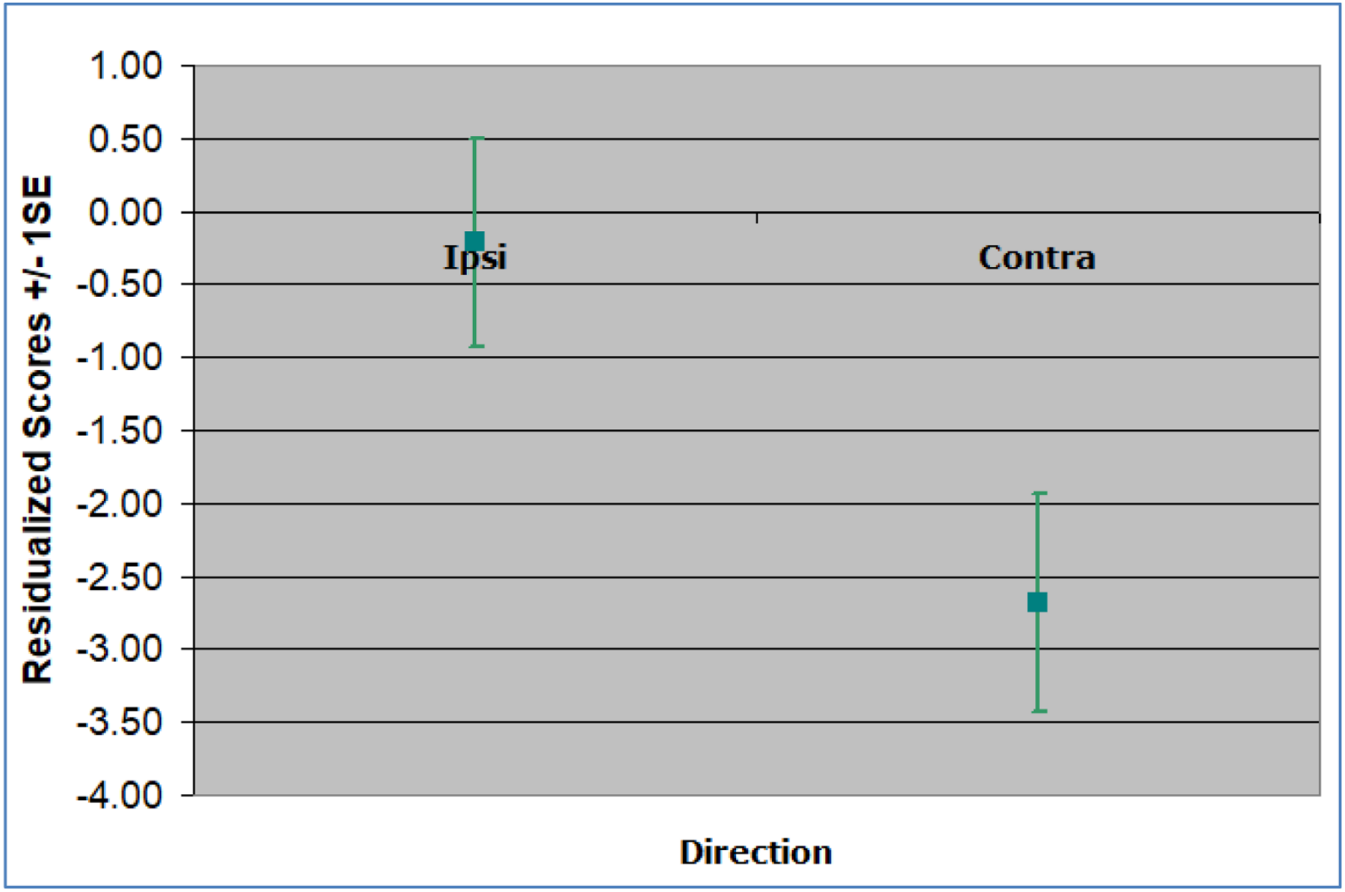
Motion Detection residualized performance scores collapsed across the three contralateral peripheral locations and the three ipsilateral peripheral locations after right frontal rTMS

Post-hoc analyses compared overall performance (an average of all eight peripheral locations) for right versus left rTMS across tasks and cortical locations. Overall TF times were slower following left parietal rTMS and faster following right parietal rTMS, t(1)=3.78, *p*=0.05 (Figure 1). MC processing was faster contralateral to rTMS than ipsilateral for right frontal stimulation, t(1)=4.10, *p*<0.05 (Figure 2). No other analyses were significant (*p*>0.05).

## Discussion

Low frequency rTMS was delivered to selected sites in the parietal, temporal and frontal cortex to disrupt processing on Troxler Fading and tasks sensitive to PC and MC processing. Low frequency rTMS can both have an inhibitory effect beneath the stimulation coil and it can disinhibit areas in projection sites distant to the stimulated brain region within and between hemispheres [25]. The first hypothesis was that impairing PC processing by stimulation of the posterior-temporal lobe would cause accelerated TF contralateral to stimulation. This hypothesis was supported following stimulation of the left hemisphere but not the right hemisphere. Texture detection was slower contralateral to left temporal stimulation and TF was faster, supporting the hypothesis that impaired PC processing accelerates TF, presumably by providing less resistance to image fading. This result is also consistent with the hypothesis that TF may be mediated primarily within a pathway sensitive to PC processing as proposed by Livingston and Hubel [4]. It is not obvious; however, why right hemisphere stimulation failed to have the same effect.

An alternative hypothesis that stimulation of the parietal cortex would impair MC processing and accelerate TF was not supported because rTMS to the parietal area did not alter MC processing. Direct stimulation of the parietal cortex, however, did alter TF. Left parietal stimulation led to slower TF times and right parietal stimulation to lead to faster TF times but it cannot be concluded that impaired MC processing was responsible. Either TF is not influenced by MC processing, or we failed to stimulate the pathway sensitive to MC processing, or the motion detection task is insensitive to MC processing. Additionally, it is possible that redundant projection pathways may compensate for any effect of parietal rTMS on MC processing. The data simply do not allow one to conclude which alternative is correct. It is interesting that a hemispheric difference is present for parietal stimulation on TF. Inhibiting neurons in left parietal cortex via low frequency rTMS can cause disinhibition of neurons in a homologous region of the right hemisphere via callosal projections [25]. Left hemisphere stimulation might slow TF (i.e., make images more resistant to fading) because it “activates” the right parietal region. Correspondingly, inhibiting the right parietal cortex with rTMS lead to faster image fading. Further, the magnitude of the effect for parietal rTMS in the right hemisphere was greater than that in the left hemisphere. This could suggest that the right hemisphere plays a dominant role with regard to TF. As mentioned above, the right parietal lobe is also a critical lesion site that causes neglect and rTMS stimulation of the right parietal lobe can mimic neglect in normal subjects. Anecdotally, our experience in examining TF in patients with neglect has been that they cannot do the TF paradigm. Most typically, patients with neglect initially report seeing the image in peripheral space but they do report fading of the image – not when it actually occurs in their experience and not when the image is made to disappear experimentally. They simply fail to report the image fading. So, it is quite possible that the parietal lobe of the right hemisphere does play an important role in TF but the current experiment does not reveal that role.

The hypothesis that frontal lobe stimulation would retard or prevent TF and make PC and MC processing more efficient was partially supported for the MC processing task. Again, a hemispheric effect was present showing an effect for the right but not the left hemisphere. Whereas DLPF rTMS did not alter TF or performance on the TD task; stimulation of the right frontal lobe did alter performance on the MD task in a systematic fashion. Motion detection was faster (i.e., more efficient) contralateral to stimulation than ipsilateral to stimulation. It is important to note that ispsilateral hemispatial performance was slower but not different from healthy/pre-rTMS performance. Damage to the DLFC frontal cortex can releases the parietal cortex from inhibition via intrahemispheric projections. Low frequency rTMS to the right DLFC may have released parietal cortex in the same hemisphere from inhibition. This could simultaneously lead to faster performance on the motion detection task contralateral to stimulation via release of the right parietal area from frontal inhibition. As mentioned above, direct stimulation of parietal cortex may have failed to disrupt performance on the MD task because the wrong area of the parietal cortex was stimulated. The DLPF is a convergence zone with reciprocal connections to other convergence zones like the junction between temporo-parieto-occiptal (TPO) region that is strongly linked to motion processing. We avoided this area to get greater specificity on MC processing and due to the overlap with the PC pathway in the TPO region [34]. If the junction had been chosen for direct stimulation it may have disrupted performance on the motion detection task.

Results indicating that rTMS of the left, posterior temporal region led to impaired PC processing are also consistent with Schiller and colleagues [5] work indicating that texture detection is specific to at least the early processing stage of the PC pathway. Our results indicate as well that the where pathway may demonstrate specificity for texture discrimination beyond striate cortex. Finally, these data are also consistent with the hypothesis that TF may occur within a pathway sensitive to PC processing.

Results of rTMS to the right posterior parietal cortex are consistent with previous research that showed accelerated TF in patients with parietal lesions [41]. One difference is the rTMS study showed opposite effects for left and right parietal stimulation, whereas the lesion study showed accelerated fading after damage to the parietal lobes of both hemispheres. A second difference is that the rTMS study failed to reveal an effect of frontal lobe stimulation on TF; whereas lesions to the frontal lobe consistently retarded or prevented image fading, again in both hemispheres. These differences almost certainly relate to the potency of rTMS for disrupting behavior versus that for structural brain injury. The effect size associated with low frequency rTMS on behavior is obviously much smaller than that associated with stroke. The value of following a stroke study with an rTMS study lies in replicating effects due to stroke in subjects who do not have all the complications associated with stroke and in suggesting new possibilities such as the lateralized differences that were observed in the present study. For example, the left hemisphere appeared to play a greater role than the right in PC processing whereas the right hemisphere appeared to have a greater role in MC processing and on TF in general. These findings are consistent with theories that posit processing specialties between the left and right hemisphere such as local-global, part-whole, and serial and parallel processing. The results of this study uniquely demonstrate the interplay between PC processing and TF and of the hemispheric laterality effects on both TF and tasks designed to be sensitive to PC and MC processing.

## Figures and Tables

**Table 1.** Subject Demographics, Visual Diagnostics, and IQ

Subjects	Male	Female
N	7	2
Age	24 (2)	23 (1)
Education	18 (1)	17 (1)
Visual Acuity	20/20 (0)	20/20 (0)
Contrast Sensitivity	1.9 (0.2)	1.9 (0.1)
Estimated FSIQ	112 (5)	116 (6)
